# Interchangeability of Sodium and Potassium Result Values of Arterial Blood Gas with Laboratory Analyzer: Narrative Review

**DOI:** 10.5005/jp-journals-10071-23110

**Published:** 2019-01

**Authors:** Yasemin Ustundağ, Kağan Huysal, Şeyda E Ozgunay, Ali R Turkoğlu

**Affiliations:** 1,2 Department of Clinical Biochemistry, University of Health Sciences, Bursa YuksekIhtisas Training and Research Hospital, Bursa, Turkey; 3 Department of Anestesiology and Reanimation, University of Health Sciences, Bursa YuksekIhtisas Training and Research Hospital, Bursa, Turkey; 4 Department of Urology, University of Health Sciences, Bursa YuksekIhtisas Training and Research Hospital, Bursa, Turkey

**Keywords:** Analyser, Ion-selective electrode, Potassium, Sodium

## Abstract

The major extracellular electrolytes, sodium, and potassium are often requested together and form a large percentage of the requested tests in routine clinical chemistry laboratories. Two types of devices that use direct and indirect ion-selective electrode (ISE) methods are used in hospitals for electrolyte measurements: blood gas analyzers (BGA), which use direct ISE technology, and the indirect ISE method, which is often used in a central-laboratory autoanalyzer (AA).

We aimed to summarize the current scientific knowledge based on whether the electrolyte test results, using Na and K test results obtained with BGA and an AA, can be used interchangeably.

We searched Medline (PubMed), Google Scholar, and Web of Science up to 31st March 2018. In addition, references of the included studies were also examined.

Fourteen studies with a risk of bias were included in the analysis. Limits of agreement differences were variable among BGA and AA sodium and potassium test results in clinical practice.

The results of both BGA and AA measures should not be used interchangeably under the assumption that they are equivalent to each other.

**How to cite this article:**

Ustundağ Y, Huysal K, Ozgunay ŞE, Turkoğlu AR. Interchangeability of Sodium and Potassium Result Values of Arterial Blood Gas with Laboratory Analyzer: Narrative Review. Indian Journal of Critical Care Medicine, January 2019;23(1):35-42.

## INTRODUCTION

The major extracellular electrolytes, sodium, and potassium are often requested together and form a large percentage of requested tests in routine clinical chemistry laboratories.

Sodium is responsible for the osmotic pressure of the extracellular fluid, and the physiological level in serum is 135 to 145 mmol/L.^1^ Potassium is the major cation in intracellular fluid and, despite playing a physiological role in such processes as a contraction of skeletal muscles, it is necessary for normal cell function.^1^ The physiological serum concentration of potassium is 3.5 to 5.0 mmol/L.^1^ Electrolyte disorders constitute a significant proportion of emergency department (ED) visits and are also common in the intensive care unit (ICU) patients and critically ill patients.^2-6^ Hyponatremia, defined as a sodium concentration <135 mmol/L, is the most common electrolyte abnormality encountered in the emergency room and ICU and can lead to serious neurological complications.^3,4^ Hyperkalemia, which occurs with potassium higher than 5.0 mmol/L, is a common electrolyte disorder leading to morbidity and mortality due to cardiac dysrhythmia, especially in ED patients.^5^

Because abnormal serum sodium and potassium levels are associated with mortality and morbidity, it is extremely important for patients to be diagnosed quickly and to start treatment early.^6^

### Laboratory Diagnosis of Electrolyte Disorders

Electrolytes are routinely measured by either direct or indirect ISE. The principle of the method is based on the determination of the electromotive power (potential) changes occurring between the measuring electrode and the reference electrode, whereas the ion to be measured interacts with the ISE membrane.7

Two different types of devices using direct and indirect ISE methods are used in hospitals for electrolyte measurements. BGA use direct ISE technology that measures electrolytes in undiluted sample types. Devices based on the indirect ISE method are often used in a high-efficiency central laboratory AA. Before measuring electrolyte concentrations with the indirect ISE method, the same diluent volume is used by estimating the amount of dilution by the expected solid fraction (7%). However, if the solid fraction is increased, as, during hyperproteinemia, the measured ion concentration is underestimated because of the higher dilution.^8^

Electrolyte values of the venous blood samples can be obtained after an average of 60 minutes in the AA in routine clinical chemistry laboratories. In the meantime, treatments depending on electrolyte values are required to be performed immediately, and are either done blindly or are delayed.^9^ In addition, the result times are even longer when the samples are hemolyzed, lipemic, inadequate, or lost, and while the devices are in the process of calibration. BGAs are especially advantageous in such places as emergency service units and ICUs because all measurement results are available in as little as 2 minutes. However, in routine clinical practice, BGA electrolyte findings are generally used to support diagnoses; that leads to a comparison of these device outputs to the AA results.^10-12^ With the observation of differences between the two results (BGAversus AA), even if samples are taken at the same time, physicians are often faced with the question of which test result to use in patient treatment, especially when therapy is to be initiated or frequent measurements are made to guide treatment.

We aimed to find whether the electrolyte test results using Na and K test results obtained with BGA and AA could be used interchangeably.

## METHOD

This article is neither a systematic review nor a meta-analysis. We searched Medline (Pubmed), Google Scholar, and Web of Science for English only in humans up to 31st March 2018; using the query 'blood gas analyzer or autoanalyzer in combination with sodium and/or potassium'. To eliminate as much unrelated research as possible, we determined that keywords must appear in the title or abstract. In addition, to include as many related studies as possible, references of the included studies were also examined. We then selected the publications with potential practical usefulness. We kept studies of adults but not children.

We excluded studies that use correlation and regression analysis, or the difference between the averages instead of Bland-Altman methods. The Bland-Altman plot quantifies the bias and a range of agreement within which 95% of the differences of the second method (as compared to the first one) fall. The Bland-Altman method objectively measures the differences between measurement techniques.^12,13^

The acceptability criteria of interchangeability of results were derived from The United States Clinical Laboratory Improvement Amendments (US CLIA) guidelines, which state that 95% of results should fall within 0.5 mmol/L for potassium levels and 4 mmol/L for measured sodium levels to assess the intralaboratory quality of clinical chemistry tests.^13,14^

## RESULTS

There are some methodological problems when comparing BGA and AA electrolyte results. For example, some studies used the results of the heparinized arterial sample is plasma and the venous sample is serum the patient's database retrospectively, whereas others compared prospective sample results. As this may account for some of the differences.

### Some Prospective Studies Investigate Whether the Electrolyte Test Results Using Arterial Whole Blood Versus Arterial Plasma or A Serum Specimen can be Used Interchangeably

In some of these prospective studies, the mean bias for Na was 1.3 to 1.7 mmol/L and for K 0.2 to 0.3 mmol/L, which are acceptable ranges using analytical goals defined by CLIA.^15-17^ However, 95% limits of agreement (LOA) were between -9.4 and 12.6 mmol/L for Na and -0.58 and 1.24 mmol/L for K, and interchangeable use is unacceptable.^15,16^

In these studies, the devices of different manufacturers were compared (Table 1). Yilmaz et al. compared the results of the Abbott C 8000 Architect AA (Abbott Diagnostics, Abbott Laboratories, North Chicago, Illinois, USA) and Siemens Rapid Point 500BGA (Siemens Health-care Diagnostics, Inc. Tarrytown, NY, USA).^15^ King et al. compared the results of the electrolytes with a Hitachi 717AA (Boehringer Mannheim, Lewes, West Sussex, UK) and a Radiometer ABL 505 BGA (Radiometer, Crawley, West Sussex, UK).^16^

Allardet-Servent et al. demonstrated that 48% of the differences between the electrolyte test results of the two analyzers-an AU 580 AA (Beckman Coulter, Brea, CA, USA) and a RAPID Point 500 BGA(Siemens Healthcare Diagnostics Inc., Tarrytown, NY, USA)-were due to changes in the serum protein level.^17^

Chacko et al. compared the sodium results of whole blood and serum samples; the mean bias was -4.07 mmol/L and 95% LOA -8.8 to 0.7.^18^ They used a GEM 3000 BGA (Instrumentation Laboratory, Werfen, Italy) and an Olympus AU2700 AA (Olympus Optical Company, Ltd., Japan). The mean difference in potassium values was -0.3 mmol/L and 95% LOA -0.72 to +0.13 mmol/L. However, individual differences were clinically significant, especially at low potassium levels (<3.0 mmol/L), and they suggested that delivery to the central laboratory by a pneumatic transport system may lead to hemolysis.^18^

**Table 1:** Summary of the studies

*Reference*	*Number of samples*	*Sampling and type of tubes*	*ABG analyzer*	*Autoanalyzer and sample type*	*Study design*	*Bland-Altman Results*	*Comment*
					Arterial whole blood versus arterial plasma or serum		
King et al. 2000; UK	115 paired critical ill patients	Samples were taken from an indwelling arterial cannula to preheparinised syringe (Drihep-PlusÒ, Becton Dickinson Acutecare, Franklin Lakes, NJ, USA)	Radiometer ABL 505 (Radiometer, Crawley, West Sussex, UK)	Hitachi 717 (Boehringer Mannheim, Lewes, West Sussex, UK) Clot activated serum seperator tube	Prospective	Na mean bias 1.7 mmol/L (%95 LOA -2.9 to 6 mmol/L) K mean bias 0.2 (%95 LOA - 0.4 to 0.8 mmol/L)	Serum analyzed within 2 hours.
Yılmaz, et al. 2016; Turkey	100 critical ill patients	Samples were taken from BD A-LINE arterial line blood gas collection syringe, (Becton, Dickinson Diagnostics®, Plymouth, UK) coated with 80 I.U Ca-heparin.	Siemens Rapid Point 500 (Siemens Healthcare Diagnostics Inc. Tarrytown, NY, USA)	Abott C 8000 Architect (Abbott diagnostics, Abbott Laboratories, North Chicago, Illinois, USA) Non-additive silicone coated tube	Prospective	Na mean bias 1.6 mmol/L (%95 LOA - 9.4 to 12.6 mmol/L) K mean bias 0.33 (%95 LOA - 0.58 to 1.24)	Serum analyzed within 1 hour.
Allerdet -Servent et al. 2017; France	314 critical ill patients	Samples were taken from indwelling arterial cateter to preset heparinised syringe.	Siemens RAPID Point 500 (Siemens Healthcare Diagnostics Inc. Tarrytown, NY, USA)	AU 5800Beckman Coulter (Beckman Coulter, Brea, CA, USA). Serum taken after 5 ml arterial blood withdrawnto BD-vacutainer tubes.	Prospective	Na mean bias 1.3 mmol/L(%95 LOA - 2.2 to 4.8mmol/L) K mean bias 0.20 (- 0.18 to 0.58 mmol/L)	Serum analyzed within 2 hours. % 48 of the difference between two analysers due to change in serum protein level.
Chacko et al. 2011; India	44 critical ill patients	Samples were taken from arteria with a DRIHEP A-LINE arterial blood gas collection syringe, (Becton Dickinson Diagnostics®,)	GEM 3000® ABG analyzer (İnstrumentation Laboratory,Werfen, Italy)	Olympus AU2700 discrete chemistry analyzer (Olympus Optical Company, Ltd., Japan) nonadditive tubes(Plymouth, UK) -Serum	Prospective	Na mean bias minus 4.07mmol/L ( 95% LOA -8.8 to 0.7) Potasyum mean bias -0.3mmol/L(% 95 LOA -0.72to 0.13)	Delivery to central laboratory by pneumatic transport system
Auvet et al. 2016; France	491 patients Cardiac Surgery Operating Room, and ICU	Samples were taken from arterial cannula (Blood management system VAMP™ 60 in with Armmount Reservoir, Edwards Lifesciences^™^, Irvine, USA) heparinized syringe	ABL 825® FLEX analyzer adiometer,Copenhagen, Denmark).	AU 2700 AA (Beckman Coulter Inc., Miami, FL, USA). Serum- non additive tubes(BD Vacutainer®, Le Pont de Claix, France)	Prospective	Na mean bias 1 mmol/L (%95 LOA -3 to 4) Potasyum mean bias 0.1mmol/L (%95 LOA -0.1 to 0.5)	Almost perfect preanalytical conditions
Jose et al. 2008; UK	529 paired results of BGA and arterial AA measurements of potassium in 121 critically ill patients		Bayer Rapidlab 865 (Siemens Healthcare Diagnostics Inc. Tarrytown, NY, USA)	Olympus AU640 or Olympus AU 2700 (Beckman Coulter Inc.,CA, USA) The arterial blood sample is taken into a nonheparinised syringe, also after the first 5 ml of blood has been discarded.	Retrospective	Potasyum mean bias 0.03 mmol /L, (% 95 LOA 0.011 to 0.056)	Serum analyzed within 1 hour.
Story et al. 2007; Australia	300 critically ill patients	Arterial blood to heparinized blood-gas syringes (Rapidlyte)	Ciba corning 865 (Ciba Corning Diagnostics, Medfield, USA)	Hitachi 747 (Roche diagnosis, Sydney, Australia) . Plasma (Lithium heparin tubes)	Retrospective	Na mean bias 2.1 mmol/L (%95 LOA - 1.8 to 2.4)	Strong correlations between differences in sodium measurements and albumin.
Banerjee et al. 2018; India	100 ICU patients	Arterial blood to heparinized blood-gas syringes (Dispovan single-use syringe)	ABL 800 (Radiometer,Copenhag en,Denmark).	AU640 (Beckman Coulter Inc., Miami, FL, USA). BD vacutainer serum tube	Prospective		Serum analyzed within 2 hours. Delivery to central laboratory by pneumatic transport system
					Arterial whole blood versus venous plasma or serum		
Zhang et al. 2015; China	200 patients presented to emergency depertmant	BD preset blood gas syringes containing solid Ca2+-balanced lithium heparin were used for sampling the arterial blood.	ABL 90 FLEX blood gas analyzer (Radiometer Medical ApS, Copenhagen, Denmark)	Vt-5600 automatic biochemical analyzer (Johnson and Johnson Services, Inc. New Brunswick, New Jersey, USA)	Prospective	Sodium mean bias was 3.04 (95% LOA 2.73 to 3.34) mmol/L Potassium mean bias was 0.43 (0.29 to 1.16) mmol/L	The biases in 32 pairs of Na values surpassed the limits of US CLIA (±4mmol/l). The biases in 44 pairs of K values surpassed the limits of US CLIA (±0.5mmol/l).
Wongyingsinn et al. 2009; Thailand	65 paired results from 53 patients admitted to emergency department	Arterial blood directly taken into a heparinised syringe	Bayer 348 (Siemens Healthcare Diagnostics Inc., Tarrytown, NY, USA)	Roche Modular ISE1800 Analyzer (Roche Diagnostics, Indianapolis, IN, USA)	Prospective	Potassium mean bias was 0.49 (- 0.34 to 1.33) mmol/L	A gap between paired samples that ranged between one minute and fifty-four minutes to draw blood.
Morimatsu et al. 2003; Australia	300 consecutive critically ill patients admitted to ICU	Blood-gas syringes (Rapidlyte; Chiron Diagnostics, East Walpole, MA)	Rapilab 865 (Bayer Diagnostics, Sydney, Australia).	Hitachi 747 (Roche Diagnostics, Sydney, New South Wales, Australia) Plasma Lithium-heparin tubes with gel separation (Vacuette; Greiner Labortechnik, Kremsmunster, Austria)	Retrospective	Sodium mean bias was 2.1 mM (95% limits of agreement: _2.6 to 6.8 mM) Potassium mean bias was minus 0.15 mM (95% limits of agreement:-0.82, to 0.79 mM).	
You et al. 2014; Korea	1188 patients with suspected hyperkalemia		NOVA Stat Profile CCX (Nova Biomedical, Waltham ,MA, USA)	Hidachi 7600(Hidachi, Tokyo, Japan)	Retrospective	Potassium mean bias was minus 0.15 mM (95% limits of agreement:-1.4, to 0.6 mM).	
Açıkgöz SB et al. 2016; Turkey	118 patients with acute potassium elevations		ABL 700 (Radiometer Medical ApS,Copenhagen, Denmark)	Architect c16000 (Abbott Diagnostics, Abbott Laboratories, North Chicago, Illinois, USA) Venöz tam kan	Retrospective	Potassium mean bias was 0.62 mM (95% limits of agreement:-0.22, to 1.46 mM).	
Budak et al. 2012 Turkey	84 ICU patients	Heparinized blood-gas syringes (Gaslyte, Totawa, NJ)	pHOx Stat Profile Plus L (Nova Biomedical, Waltham MA, USA)	Roche Modular ISE 900 (Roche Diagnostics, Mannheim, Germany). Arterial blood to clot-activating tubes (Green- Vac, Yongin, Korea)	Retrospective	Sodium mean bias was 2.1 mM (95% limits of agreement: - 0.97 to 10.05 mM) Potassium mean bias was minus 0.25 mM (95% limits of agreement: - 0.59, to 1.1 mM).	Samples were sent via pneumatic tube deliver system
Johnston et al. 2005; UK	50 cardiac arrest patients		IL 1640 (Instrumentation Laboratory System, Lexington, Mass., USA)	Olympus Analyser (Beckman Coulter Inc., Miami, FL, USA)	Retrospective	Potassium mean bias was 0.106 mM (95% limits of agreement:-1.81, to 1.39 mM)	Samples were sent via pneumatic tube deliver system

Auvet et al. compared 491 paired whole blood and plasma sample electrolyte levels with ABL 825® FLEX BGA (Radiometer, Copenhagen, Denmark) and AU2700 AA(Beckman Coulter Inc., Miami, FL, USA) devices. Because preanalytical conditions were near-perfect, the bias of the results obtained for potassium was 0.1 (95% LOA 0.1-0.5), and the bias for sodium was 1 (95% LOA minus 3 to 4); both results are interchangeable.^19^

In a recent study, Banerjee et al. compared ABL 800 (Radiometer, Copenhagen, Denmark) with an AU640 AA (Beckman Coulter Inc., Miami, FL, USA) analyzers to find a correction factor for the appropriation of the ABG value with the AA to minimize all errors. They concluded that a correction factor should be determined individually for each hospital.^20^

### Some of the Studies Were Retrospective, Comparing the Levels of Electrolytes Using Arterial Whole Blood Versus Arterial Plasma or Serum

Jose et al. compared critical electrolytes run on a Bayer Rapidlab 865 BGA (Siemens Healthcare Diagnostics Inc., Tarrytown, NY, USA) and an Olympus AU640 AAor an Olympus AU2700 AA (Beckman-Coulter, Inc., Fullerton, CA, USA). The difference between the potassium values of the two methods is 0.03 mmol/L and 95% LOA 0.011 to 0.056. The Bland-Altman statistical method shows that even in hypokalemia and hyperkalemia, 95% of the patient results bias is less than 0.5 mmol/L.^21^

Story et al. evaluated electrolytes with albumin levels and demonstrated that if the plasma albumin level was above 40 g/L, the bias was 0, and the indirect ISE Na value was found to be higher in hypoalbuminemia patients.^22^

### Some of the Prospective Studies Compare the Levels of Electrolytes Using Arterial Whole Blood Versus Venous Serum or Plasma

The study of Zhang et al. prospectively compared arterial and venous blood Na and K results obtained with an ABL 90 FLEX BGA (Radiometer Medical ApS, Copenhagen, Denmark) and a VT-5600AA (Johnson and Johnson Services, Inc., New Jersey, USA). The mean difference between the two devices for sodium was 3.04, and 95% LOA was minus 1.24 to 7.31 mmol/L. The paired test result biases of 32/50 were higher than the values accepted by the US CLIA. The average bias for potassium was 0.43 mmol/L (95% LOA minus 0.29-1.16 mmol/L). The 44/50 pairs of values exceeded the acceptable range for US CLIA (0.5 mmol/L).^23^

Wongyingsinn et al. studied whole blood electrolyte levels with a Bayer 348BGA (Bayer Diagnostics, Siemens Healthcare Diagnostics Inc., Tarrytown, NY, USA) and venous blood with a Roche Modular ISE 1800 AA (Roche Diagnostics, Indianapolis, IN, USA). The mean difference between the two methods was 0.49 mmol/L (95% CI of LOA 0.893-0.943). However, this difference is explained by the range of 1-54 minutes for taking blood from the patient.^24^

### Retrospective Studies Comparing the Levels of Electrolytes Using Arterial Whole Blood Versus Venous Serum or Plasma

In retrospective studies comparing the arterial whole blood and venous serum or plasma results, the mean bias for sodium was found to be 4.9 to 2.1 mmol/L, and the LOA was minus 0.97 to 10.05 mmol/L.11,25 With the comparison of potassium, Bland-Altman AA, and BGAdata, the mean bias was 0.106 to 0.4mmol,14, 28,29 95% LOA-1.4 and 1.394 mmol/L, and the two devices were not interchangeable.^25-27^

Johnston and colleagues analyzed K+ in arterial and venous blood samples taken from 50 patients during cardiac arrest; and ran them on an IL1640 BGA(Instrumentation Laboratory System, Lexington, Mass., USA). The venous sample was run for analysis via a pneumatic tube delivery system through a central Olympus AA (Beckman Coulter Inc., Miami, FL, USA). Johnston explained that the differences between the results are due to unidentified hemolysis.^27^

In their retrospective analysis, Açıkgöz and colleagues compared 118 patients with acute potassium elevations analyzed with an ABL 700 radiometer (Radiometer, Copenhagen, Denmark) with the Architect's c16000 AA (Abbott Diagnostics, Abbott Laboratories, ILL, USA). The mean difference between the two methods was 0.62 ± 0.43 mmol/L (95% confidence intervals: 1.462 and -0.222).^28^

## DISCUSSION

There are several reasons for the differences observed in BGA and AA interchangeability studies for Na and K results.

The main reason for differences in the potassium value measured by the BGA device is that the hemolysis is not noticeable in the arterial specimen. Hawkins et al. reported that hemolysis is higher than predicted, and 33% of hypokalemic cases could not be detected with BGA.^29^ Venous samples are always centrifuged and then visually examined for hemolysis in routine laboratories. When hemolysis is detected, the sample is discarded, or no measurement is made for potassium. Meanwhile, the release of K from the platelets during coagulation may cause an increase in serum.^30^

The pneumatic tube system may lead to hemolysis,31 and potassium levels may differ in the central laboratory.^18,27,32^ Also; the difference between the time elapsed between sampling and analysis can influence the BGA and AA electrolyte measurements, especially K.^24^

The use of different syringes or tubes containing the anticoagulant in sample preparations may be responsible for the preanalytical bias of the measured electrolytes in the BGA device.^33-35^ Dilution of the plasma volume of the sample with the use of conventional syringes washed with liquid heparin may cause the actual value of the BGAelectrolytes to be lower.^34-37^ In addition, heparin itself binds positively charged ions and lowers the value of the electrolytes measured.^14,38^

Studies have reported that when comparing BGA with an AA device, serum protein, and albumin levels are significantly important.^17^ The difference between the results was found to be correlated with serum albumin and total protein concentrations.^9,22^

It is important to detect small changes in the relative sodium concentrations in the follow-up of critical situations.^38^ Patients with symptomatic hyponatremia require slow correction, so patients' serum sodium levels should be monitored frequently.^39^ However, plasma protein levels may vary during treatment. In such cases, it is necessary to obtain the results from a single analyzer and minimize the analytical differences between the devices. The accuracy and stability of the different calibrators used in each autoanalyzer are important for the reliability of the results, which can lead to differences in comparison studies.^40^

### Limitations

For the measured variables, it is not possible to determine which electrolyte values (BGA *vs.* AA) are closer to the true value. Although external or internal quality-control studies are performed in routine laboratories; it does not show the real value of the sample.

## CONCLUSION

It seems inappropriate to draw a conclusion about the interchangeability of different device results. Electrolyte levels should be regularly monitored, and the results of both measures should not be used interchangeably under the assumption that they are equivalent to each other.
